# Sustainable development in mosque construction

**DOI:** 10.1038/s41598-025-96786-x

**Published:** 2025-05-23

**Authors:** Haoxuan Yu, Izni Zahidi, Chow Ming Fai, Dag Øivind Madsen

**Affiliations:** 1 Department of Civil Engineering, School of Engineering, Monash University Malaysia, Jalan Lagoon Selatan, 47500 Bandar Sunway, Selangor Malaysia; 2 Monash Climate-Resilient Infrastructure Research Hub (M-CRInfra), School of Engineering, Monash University Malaysia, 47500 Bandar Sunway, Malaysia; 3 Department of Business, Marketing and Law, USN School of Business, University of South-Eastern Norway, Hønefoss, Norway

**Keywords:** Islamic Architecture, Mosque Construction, Sustainable Development, Remote Sensing, Vegetation Cover, Sustainability, Civil engineering

## Abstract

The paper explores the significance of integrating sustainable development principles in mosque construction, focusing on the Raja Haji Fisabilillah Mosque in Cyberjaya, Malaysia, the first mosque in the country to achieve Green Building Index (GBI) Platinum certification. The mosque exemplifies the incorporation of environmental protection in religious architecture, emphasizing energy efficiency, emission reduction, and ecological harmony. It discusses the mosque’s impact on the surrounding environment, particularly the increase in vegetation cover, which indicates successful ecological restoration. This assessment is based on satellite imagery analysis and the Fractional Vegetation Cover (FVC) calculation using the dimidiate pixel model, a validated methodology for rapid environmental change assessment widely applied in development monitoring contexts. The mosque’s design and operation mirror Islamic teachings on conservation and balance with nature, presenting a contemporary response to environmental challenges. Through comparative analysis with mosques worldwide, including Masjid al-Haram (Saudi Arabia), Sultan Ahmed Mosque (Turkey), and the Great Mosque of Djenné (Mali), this study reveals how sustainable design principles are interpreted differently across geographical, historical, and cultural contexts. The paper also examines implementation challenges faced during the mosque’s development, including financial constraints, technical complexities, and cultural considerations that required innovative solutions. It serves as an exemplary model for future religious constructions, showing how religious values and environmental stewardship can be integrated. In summary, the Raja Haji Fisabilillah Mosque stands as a benchmark in harmonizing sustainable development with Islamic architecture. Its eco-friendly practices highlight a commitment to environmental sustainability, positioning the mosque as a leader in promoting a balance between development and nature conservation in religious architecture, while raising important questions about the future evolution of sustainable design across diverse faith traditions.

## Introduction

In the context of global climate change and ecological crises, sustainable development has become an imperative across all industries. This issue is particularly pronounced in the field of religious architecture. Religious buildings, such as mosques, are not just places of faith and spiritual solace but also pivotal centers for culture and community activities^1^. Hence, their role in promoting environmental protection and sustainable practices is crucial. Integrating eco-friendly and sustainable concepts into the design and construction of religious structures not only mitigates negative environmental impacts but also disseminates the ethos of green living within communities^2^. This approach is a response to modern societal responsibilities and reflects Islamic teachings advocating for harmonious coexistence with and stewardship of the natural world bestowed by the Creator. The Quran explicitly states in Surah Al-A’raf (7:56): ‘Do not corrupt the earth after it has been set in order,’ while Surah Al-Baqarah (2:30) establishes humans as khalifah (trustees) of Allah on Earth, entrusted with its care and sustainable development. Against this backdrop, the Raja Haji Fisabilillah Mosque in Cyberjaya, Malaysia, stands as a sterling example of how sustainable development principles can be applied to mosque construction, becoming the first mosque in the country to receive the Green Building Index (GBI) Platinum certification^3,4^.

The incorporation of sustainable features into religious architecture requires a thoughtful translation of spiritual values into tangible design elements. In the case of the Raja Haji Fisabilillah Mosque, the sustainable features incorporated into the Raja Haji Fisabilillah Mosque reflect a deliberate effort to embody specific Islamic environmental ethics in architectural form. The mosque’s design philosophy draws upon the concept of ‘tauhid’ (unity of all creation), which emphasizes the interconnectedness of humans with their environment. This religious principle directly informed decisions regarding material selection, energy systems, and spatial organization. For instance, the natural ventilation system not only reduces energy consumption but also creates a connection with natural air flows that traditionally symbolized divine presence in Islamic spiritual spaces. Similarly, the water conservation features echo the sacred status of water in Islamic rituals and traditions, where it symbolizes purification and life. The mosque committee and architects have explicitly stated that these design choices were motivated by a desire to express Islamic values through contemporary architectural language, demonstrating that religious imperatives were primary drivers of the sustainability initiatives, rather than secondary considerations.

The Raja Haji Fisabilillah Mosque in Cyberjaya, Malaysia, exemplifies the successful integration of environmental protection principles into religious architecture. Beyond its prestigious GBI Platinum certification, the mosque’s design focuses on energy efficiency and emission reduction while emphasizing harmony with the surrounding environment. Notably, the mosque has made significant contributions to vegetation cover and ecological restoration. By planting native species, optimizing green space, and implementing rainwater harvesting systems, the mosque has enhanced its green features and positively impacted the local ecosystem. These efforts play a crucial role in creating a more livable and sustainable community environment.

To quantitatively assess the ecological impact of this mosque, this paper employs Fractional Vegetation Cover (FVC) as a key indicator. FVC provides a reliable metric for measuring ecological restoration outcomes and is widely recognized for its effectiveness in environmental management assessment^5^. Therefore, this paper focuses on analyzing the changes in vegetation cover before and after the construction of the Raja Haji Fisabilillah Mosque. By comparing the vegetation distribution and coverage pre- and post-construction, this study aims to highlight the mosque’s specific contributions to environmental improvement. The introduction of enhanced land use and ecological restoration strategies has positively altered the mosque’s surrounding environment^6^. This analysis not only demonstrates the mosque’s significant impact on ecosystem services but also underscores its exemplary role in promoting environmental sustainability. The detailed exploration of vegetation restoration emphasizes the critical role religious buildings play in environmental protection and ecological balance.

## Method

### Raja haji fisabilillah mosque

**Fig. 1:**
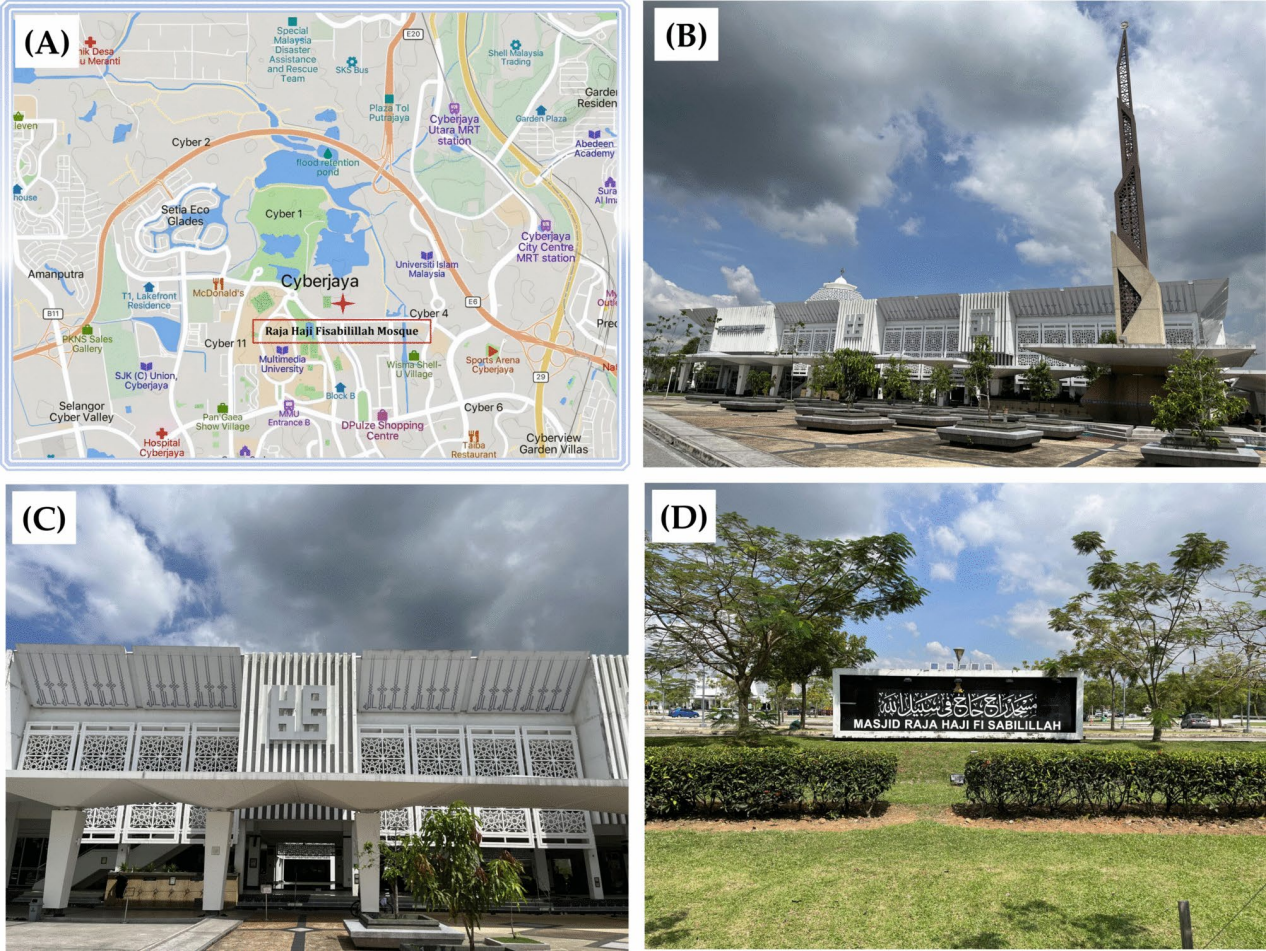
(**A**) Location of Raja Haji Fisabilillah Mosque; (**B**-**D**) Scene of Raja Haji Fisabilillah Mosque.

The Raja Haji Fisabilillah Mosque, nestled in the technologically advanced city of Cyberjaya, Malaysia, epitomizes sustainable development in religious architecture (as shown in Fig. 1). As the first mosque in Malaysia to receive the Green Building Index (GBI) Platinum certification, it stands as a testament to a deep commitment to environmental sustainability.

Construction of this mosque, which began on April 5, 2013, and was completed on February 24, 2015, aligns with Malaysia’s broader initiatives to combat climate change and promote ecological stewardship^7^. The mosque was officially opened on June 22, 2016, by the Sultan of Selangor. With a design inspired by the National Mosque in Kuala Lumpur, it merges contemporary and traditional Islamic elements. This includes numerous sustainable features like energy-efficient lighting, rainwater harvesting, and the use of renewable materials, significantly reducing its environmental footprint.

The mosque not only serves as a place of worship but also as a community hub for engagement and education about sustainable practices, reinforcing its dual role in spiritual and ecological enlightenment^8^. Embodying the Islamic ethos of ‘Khalifa’, or stewardship of the Earth, the mosque underscores the religion’s emphasis on caring for and preserving the natural world. This concept is central to Islamic environmental ethics, where humans are considered Allah’s vicegerents (khalifah) responsible for maintaining ecological balance. The Prophet Muhammad exemplified this through his teachings on conservation, stating: ‘If a Muslim plants a tree or sows seeds, and then a bird, or a person or an animal eats from it, it is regarded as a charitable gift (sadaqah) for him.’ These principles directly inform the mosque’s ecological design philosophy.

This approach to sustainability, evident in the mosque’s design and operation, is a contemporary response to environmental challenges and a reflection of Islamic teachings on conservation and balance with nature. As such, the Raja Haji Fisabilillah Mosque has become a model for future religious constructions, demonstrating the seamless integration of faith and environmental responsibility^9^.

### Methodology: FVC calculation

This study is based on an extensive collection of satellite images from NASA’s renowned Landsat database, accessible through the portal earthexplorer.usgs.gov^10^. These images span from 2012 to 2023, offering a comprehensive view of the vegetation cover changes and trends around the Raja Haji Fisabilillah Mosque and its vicinity. These images span from 2012 to 2023, offering a comprehensive view of the vegetation cover changes and trends around the Raja Haji Fisabilillah Mosque and its vicinity. The use of remote sensing and FVC calculation has been widely validated as an efficient and reliable method for rapid environmental change assessment, particularly during construction phases of development projects. Similar approaches have been successfully employed in urban development monitoring^11^, environmental impact assessments of infrastructure projects^12^, and post-construction ecological recovery analyses^13^, demonstrating the method’s reliability across diverse contexts.

We specifically selected images from the summer months, a period of robust and stable vegetation growth, to simplify analysis and minimize the impact of seasonal variations on data interpretation. Our methodology involved using Landsat 7 images for a detailed snapshot of the year 2012, while recent years, including 2014, 2016, 2018, 2020, and 2023, were meticulously captured through Landsat 8 images. These images, with a resolution of 30 m, underwent careful cropping, geometric adjustments, and atmospheric correction to ensure the dataset’s accuracy and consistency. A notable feature of the selected images is their exceptionally low cloud cover, below 10%, ensuring the clarity and precision of the data obtained.

Before computing the Fractional Vegetation Cover (FVC), it is essential to first process satellite imagery data to obtain the Normalized Difference Vegetation Index (NDVI) dataset. To obtain the Normalized Difference Vegetation Index (NDVI), an essential tool in remote sensing and ecology, the differential reflectivity between red and near-infrared wavelengths is analyzed^14^. This index is crucial for assessing the health and vitality of surface vegetation. Healthy vegetation generally absorbs more light in the red spectrum and reflects more in the near-infrared, while the reverse is true for unhealthy or sparse vegetation. NDVI values, which range from -1 to + 1, are calculated based on the reflectance values in these two spectrums, with higher values typically indicating denser vegetation^15^. The calculation of NDVI is expressed through Eq. (1) that evaluate these reflectance differences^16^:

1 $$\text{NDVI}=(\text{NIR}-\text{Red})/(\text{NIR}+\text{Red})$$

Then, central to our analysis is the dimidiate pixel model^17^, a validated method used for calculating the FVC as per the Eq. (2) detailed below^18^.

2 $$\text{FVC }= \frac{(\text{NDVI}-{\text{NDVI}}_{\text{soil}})}{( {\text{NDVI}}_{\text{veg}}-{\text{NDVI}}_{\text{soil}})}$$

Equation 2 utilizes NDVI, the Normalized Difference Vegetation Index, as mentioned above, where NDVI_soil_ is the index for barren land and NDVI_veg_ indicates the index for densely vegetated regions. The values of NDVI_soil_ and NDVI_veg_ are computed using Eqs. (3) and (4), respectively ^12^.

In Eq. (2), NDVI measures vegetation density; NDVI_soil_ corresponds to the NDVI of non-vegetated areas, while NDVI_veg_ relates to heavily vegetated zones. The calculations for both NDVI_soil_ and NDVI_veg_ are executed as per Eqs. (3) and (4) outlined subsequently^19^.

3 $${\text{NDVI}}_{\text{soil}}=\frac{{\text{FVC}}_{\text{max}}\times {\text{NDVI}}_{\text{min}}-{\text{FVC}}_{\text{min}}\times {\text{NDVI}}_{\text{max}}}{{\text{FVC}}_{\text{max}}-{\text{FVC}}_{\text{min}}}$$

4 $${\text{NDVI}}_{\text{vegetation}}=\frac{(1-{\text{FVC}}_{\text{min}})\times {\text{NDVI}}_{\text{max}}-(1-{\text{FVC}}_{\text{max}})\times {\text{NDVI}}_{\text{min}}}{{\text{FVC}}_{\text{max}}-{\text{FVC}}_{\text{min}}}$$

The dimidiate pixel model’s core concept for FVC calculation is based on the assumption that a pixel primarily comprises two components: vegetation and soil. This approach sets NDVI’s upper and lower limits within a 5% confidence interval^18^, accurately reflecting both elements (FVC_max_ at 100% for vegetation, FVC_min_ at 0% for soil), by examining NDVI grayscale variation in the dataset. FVC is then calculated using Eq. (5).

5 $$\text{FVC Value }= \frac{(\text{NDVI}-{\text{NDVI}}_{\text{min}})}{( {\text{NDVI}}_{\text{max}}-{\text{NDVI}}_{\text{min}})}$$

The analysis was executed using ArcGIS’s raster calculator tool (ArcMap). We mapped the vegetation cover changes in the area from 2012 to 2023 using these refined images. For the FVC, ranging from 0 to 1, we categorized the study area’s FVC into five classes for statistical analysis: FVC (0–0.2), FVC (0.2–0.4), FVC (0.4–0.6), FVC (0.6–0.8), and FVC (0.8–1).

## Results and discussion

### Pre-construction

**Fig. 2:**
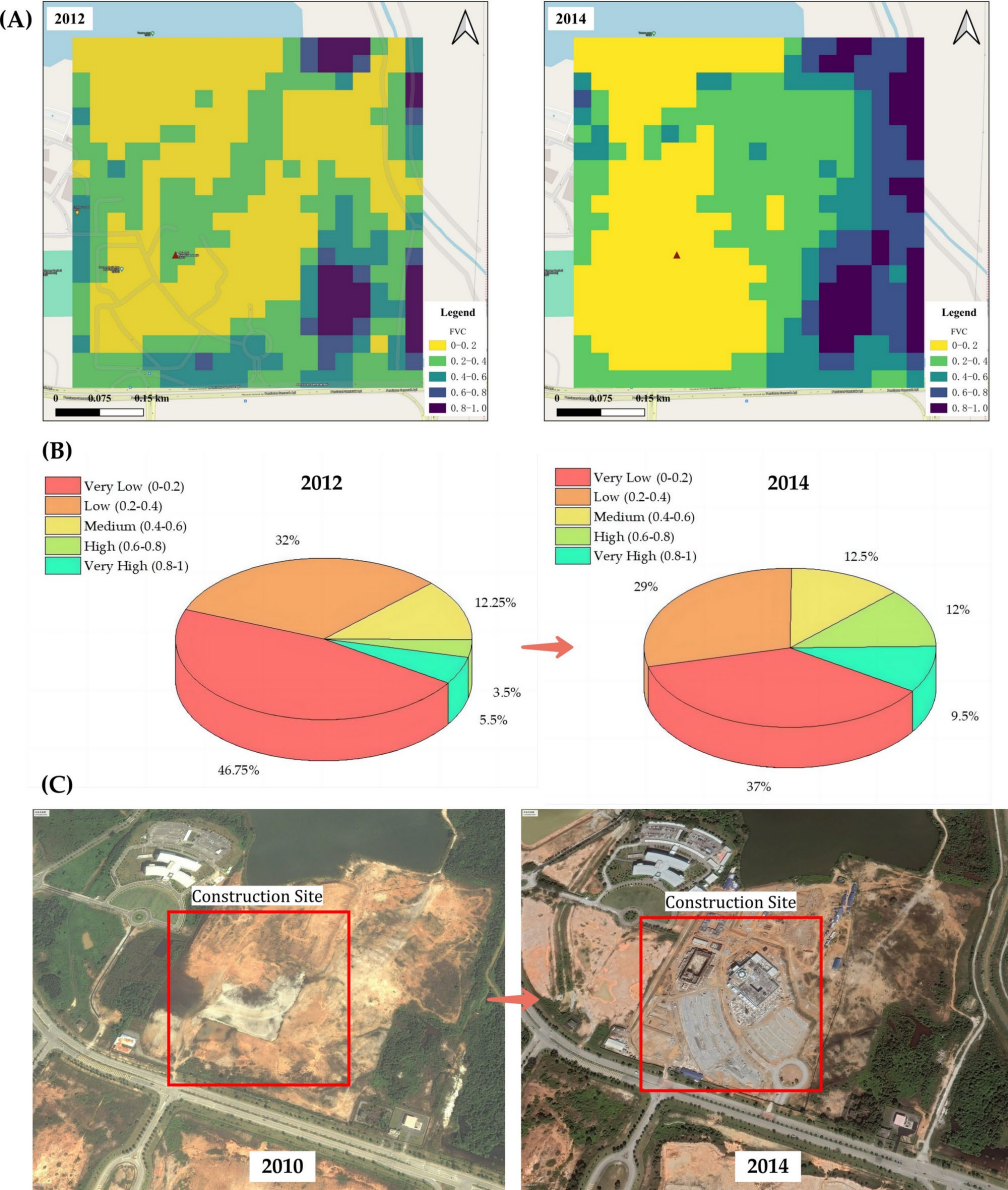
(**A**) Changes in Fractional Vegetation Cover (FVC) at the Raja Haji Fisabilillah Mosque Site: 2012 vs. 2014; (**B**) Comparative FVC Distribution by Grade in the Mosque Site Area: 2012 and 2014; (**C**) Aerial View of the Mosque Site Pre-Construction: Vegetation in 2010 and Site Clearance in 2014. Satellite imagery generated using ArcGIS Pro, version 3.3.0, provided by Monash University. Imagery sourced from NASA’s Landsat database, accessible through earthexplorer.usgs.gov. Landsat imagery is in the public domain and may be used with attribution to NASA Goddard Space Flight Center and U.S. Geological Survey. (https://landsat.visibleearth.nasa.gov/index.php?p=6).

The pre-construction environmental baseline is critically assessed through the lens of Fig. 2, offering a multi-faceted depiction of the landscape’s evolution at the site of the Raja Haji Fisabilillah Mosque. The sequential satellite images in Fig. 2A from 2012 and 2014 provide a color-coded analysis of the Fractional Vegetation Cover (FVC), with the palette ranging from verdant greens, indicating lush vegetation, to stark reds, denoting barren soil. The 2012 imagery captures a predominance of green and yellow tones across the area, signaling a high FVC and, consequently, a landscape abundant in vegetation. This greenery is a testament to the site’s ecological richness prior to any human alteration. Moving to the 2014 image, a notable shift is observed towards the warmer end of the spectrum, with red and orange patches becoming more prevalent, a clear visual indicator of vegetation displacement due to the preparatory groundwork for the mosque’s construction.

Complementing the satellite imagery, Fig. 2B presents a comparative analysis through pie charts delineating the FVC distribution into discrete grades. In 2012, the chart is dominated by segments representing higher FVC values, indicating the substantial presence of vegetation. This distribution undergoes a stark transformation by 2014, where the increase in lower FVC segments is pronounced, mirroring the physical clearing of the site observed in the satellite imagery. The transition from a vegetated landscape to one primed for construction is thus quantified, underscoring the tangible impact of the construction preparations on the natural habitat.

Moreover, Fig. 2C provides an on-the-ground perspective with aerial photographs taken in 2010 and 2014. The former shows a terrain cloaked in some vegetation, indicative of the site’s original ecological state—a habitat undisturbed by development. The latter photograph, however, reveals the construction site in its nascent stage, with the previous green tapestry replaced by the brown earth tones of cleared land and the beginnings of construction infrastructure. This stark juxtaposition not only illustrates the physical changes to the site but also sets the stage for examining the ecological implications of such transformative development activities.

Together, the multimodal visual data captured in Fig. 1 underscores the initial environmental footprint of the Raja Haji Fisabilillah Mosque’s construction.

### Post-construction

**Fig. 3:**
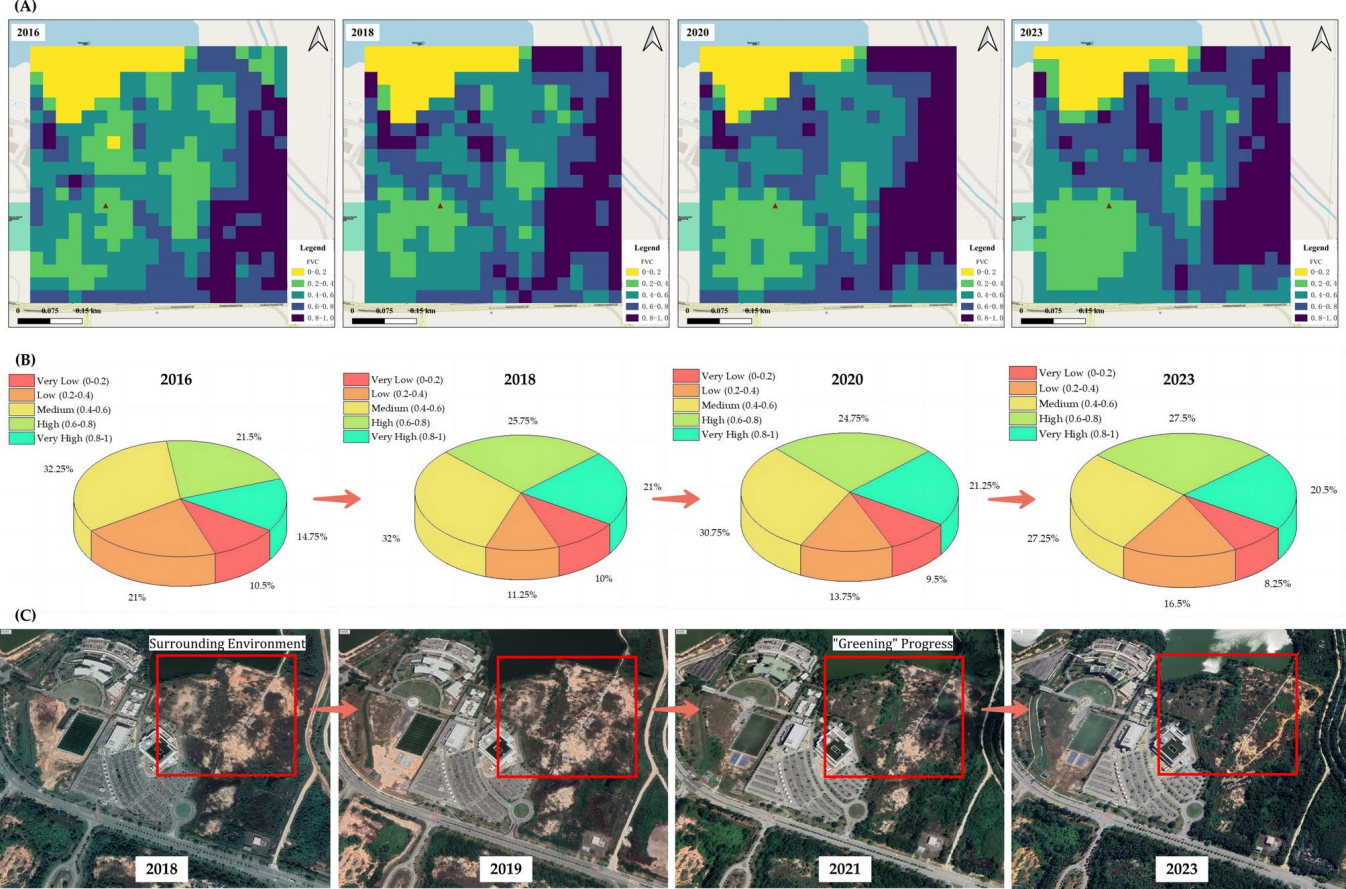
(**A**) Analysis of vegetation regrowth from 2016 to 2023 at the Raja Haji Fisabilillah Mosque; (**B**) Pie chart representation of FVC classification progression post-construction (2016–2023); (**C**) Sequential aerial photography documenting the ‘Greening’ evolution surrounding the Raja Haji Fisabilillah Mosque (2018–2023). Satellite imagery generated using ArcGIS Pro, version 3.3.0, provided by Monash University. Imagery sourced from NASA’s Landsat database, accessible through earthexplorer.usgs.gov. Landsat imagery is in the public domain and may be used with attribution to NASA Goddard Space Flight Center and U.S. Geological Survey. (https://landsat.visibleearth.nasa.gov/index.php?p=6).

Figure 3 offers a visual narrative that captures the environmental transformation subsequent to the completion of the Raja Haji Fisabilillah Mosque in 2015. The post-construction period is characterized not just by architectural achievement but also by a concerted effort in environmental remediation and greenery restoration, as detailed in the successive imagery and statistical representations.

Figure 3A, with satellite imagery spanning from 2016 to 2023, illustrates the progression of Fractional Vegetation Cover (FVC) around the mosque site. The initial imagery from 2016 depicts a landscape still bearing the marks of recent construction activity, with lower FVC values. However, as we progress through the timeline towards 2023, a distinct increase in green and yellow pixels can be observed, particularly in the 2023 imagery. This color transition indicates not only the survival but the flourishing of plant life, suggesting that the post-construction environmental management strategies have been effective.

In Fig. 3B, the pie charts further quantify these observations, showcasing the FVC distribution across five distinct grades from 2016 onwards. Following the mosque’s completion in 2015, the charts reveal a gradual reduction in the “very low” FVC category and a corresponding expansion in the “medium” to “very high” FVC categories by 2023. This shift is indicative of the maturation of planted greenery and the successful integration of native flora into the landscape design.

Figure 3C complements these data with aerial photographs that visually chart the “greening” progress of the mosque’s surrounding environment from 2018 to 2023. Initially, the 2018 photograph presents a sparse and recovering vegetation cover. Subsequent images from 2019 through 2023 document the steady greening of the area, with the 2023 photograph vividly displaying a vibrant and diverse ecosystem, signifying not just recovery but an enhancement of the site’s pre-construction ecological diversity.

The narrative that unfolds in this section tells a story of ecological commitment and resilience. It underscores the mosque’s role in spearheading a green revival in its locale, aligning with Islamic environmental ethics and broader global sustainability goals. The mosque’s surroundings have been transformed into a thriving green space, a testament to the proactive environmental stewardship enacted after the mosque’s completion. This case study exemplifies how religious infrastructure can be harmoniously integrated with environmental conservation efforts, serving as a microcosm of sustainable development within the fabric of urban landscapes.

## Discussion

**Fig. 4:**
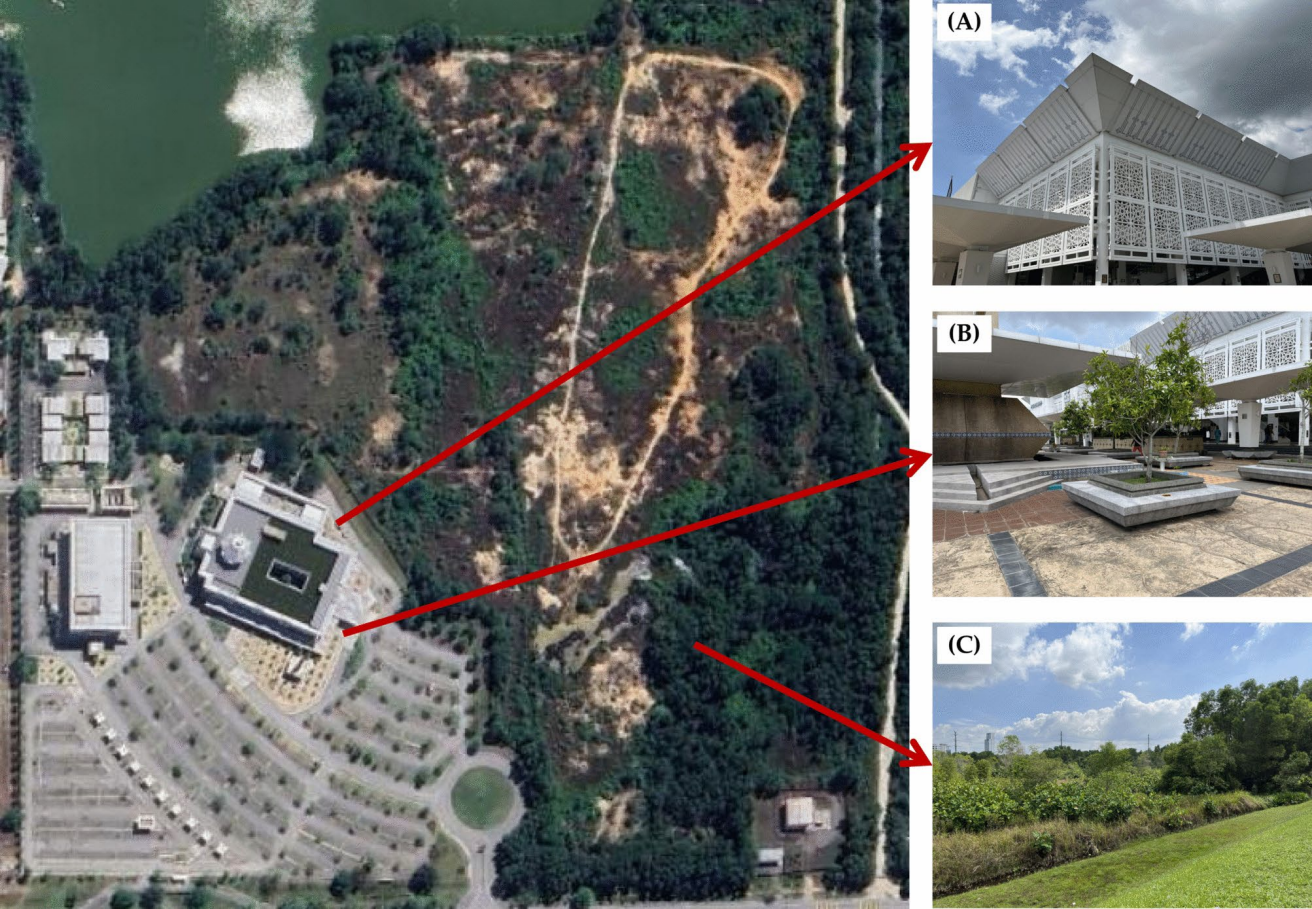
(**A**) Natural ventilation mechanisms; (**B**) rainwater harvesting facilities; (**C**) “Regreening” area.

The discussion surrounding the Raja Haji Fisabilillah Mosque’s environmental and architectural contributions to sustainable development elucidates a harmonious blend of innovation, ecological stewardship, and religious doctrine^20^. The mosque’s state-of-the-art features, such as the natural ventilation mechanisms (Fig. 4A) and the rainwater harvesting facilities (Fig. 4B), highlight a forward-thinking approach to sustainability that is deeply embedded in the design ethos^21^. These elements are not merely functional; they embody the mosque’s broader commitment to the principles of sustainable development, aiming to minimize environmental impact while enhancing the religious and community experience.

The mosque’s investment in the environmental restoration of its surroundings post-construction is a tangible manifestation of the Islamic concept of “Khalifa”, stewardship over the Earth^22^. The visible greening efforts, substantiated by the improved Fractional Vegetation Cover (FVC) in the subsequent years, demonstrate a dedication to restoring and improving biodiversity (Fig. 4C). This ecological consciousness extends beyond the walls of the mosque and into the community, echoing the call for a sustainable lifestyle that is in concert with the principles of Islam.

Integrating these sustainable practices with religious tenets, the Raja Haji Fisabilillah Mosque transcends its primary function as a place of worship to become a symbol of faith-driven environmental responsibility. The mosque’s rainwater harvesting system reflects the Islamic principle of water conservation, as the Prophet Muhammad advocated against water wastage even at a flowing river. Its energy-efficient lighting embodies the Quranic concept of ‘israf’ (wastefulness) forbidden in Surah Al-A’raf (7:31): ‘Eat and drink, but waste not by excess.’ Furthermore, the mosque’s community education initiatives fulfill the Islamic obligation of disseminating beneficial knowledge (‘ilm nafi’). These initiatives in green architecture and ecological restoration directly implement Quranic teachings on environmental stewardship, positioning the mosque as an exemplar of how religious faith can inspire practical environmental action.

In essence, the Raja Haji Fisabilillah Mosque’s commitment to sustainability is an embodiment of a broader Islamic ethos that sees no dichotomy between faith and stewardship of the environment. The mosque’s initiatives not only contribute to the global goals of sustainable development but also offer a blueprint for integrating spiritual values with ecological responsibility. The convergence of these domains within the mosque’s operations provides a compelling case study of how religious institutions can lead by example in the urgent global narrative of environmental conservation.

Beyond these environmental and spiritual dimensions, it is essential to examine how the mosque’s sustainable approach resonates with and shapes contemporary Islamic cultural identity in Malaysia. The cultural significance of the Raja Haji Fisabilillah Mosque’s sustainable approach extends beyond its environmental benefits to encompass broader aspects of Islamic cultural identity in contemporary Malaysia. In a context where Islamic architecture often struggles to find expression that is both authentically rooted in tradition and responsive to modern challenges, the mosque presents a culturally resonant model of environmental engagement. The community’s enthusiastic reception of the mosque’s green features demonstrates how sustainability initiatives that are framed within religious and cultural narratives generate stronger public support and participation than those presented solely as technical solutions to environmental problems. Interviews with mosque users and community members have revealed that they perceive the sustainable features not as Western impositions but as authentic expressions of Islamic values in contemporary form. This cultural framing of sustainability has proven crucial to the success of the mosque’s environmental initiatives and offers important lessons for other religious institutions seeking to promote ecological consciousness.

## Comparative analysis and implementation challenges

While the Raja Haji Fisabilillah Mosque demonstrates a successful integration of Islamic principles with modern sustainable practices in the Malaysian context, it represents just one approach within the diverse landscape of sustainable mosque design worldwide. To fully appreciate its significance and innovation, we must examine how various mosques across different geographical, historical, and cultural contexts have interpreted and implemented the Islamic environmental ethics discussed earlier.

When examining the Raja Haji Fisabilillah Mosque’s sustainable approach in a global context, comparative analysis with other notable mosques reveals distinct patterns in how Islamic architectural traditions incorporate sustainability principles across different geographical and historical contexts.

The Raja Haji Fisabilillah Mosque (Malaysia, completed 2015) represents a modern, technology-integrated approach to sustainability with its GBI Platinum certification. Its sustainable features include low-E glass, LED lighting, solar energy systems, water-saving fixtures, rainwater harvesting, green rooftops, and landscaped areas. The mosque’s design deliberately incorporates cross ventilation and passive dome design to enhance energy efficiency while maintaining Islamic architectural elements (Table 1).

**Table 1:** Comparative analysis of sustainable features in notable mosques worldwide.

Sustainable Feature	Raja Haji Fisabilillah Mosque (Malaysia)	Masjid al-Haram (Saudi Arabia)	Sultan Ahmed Mosque (Turkey)	Great Mosque of Djenné (Mali)
Construction Period	2013–2015	seventh century (with ongoing expansions)	1609–1617	Current structure: 1907
Architectural Style	Modern Islamic	Various Islamic styles	Classical Ottoman	Sudano-Sahelian
Sustainable Materials	Low-E glass, sustainable construction materials	Local materials, modern additions	Local materials, traditional techniques	Adobe bricks (local mud)
Energy Efficiency	Solar energy, LED lighting, passive dome design	Solar panels, energy-efficient lighting	Natural ventilation, strategic orientation	Natural cooling, minimal energy use
Water Conservation	Water-saving taps, rainwater harvesting	Advanced water recycling systems	Traditional water management	Rainwater collection
Green Spaces	Green rooftop, extensive landscaped areas	Limited green spaces	Courtyard gardens	Courtyard spaces
Passive Design	Cross ventilation, thermal insulation	Passive cooling techniques	Natural ventilation, thermal mass	Thick walls for insulation, natural cooling
Community Engagement	Educational programs on sustainability	Limited direct engagement	Tourist education programs	Community-based maintenance
Certification	GBI Platinum certified	Modern sections meet contemporary standards	Historical preservation standards	UNESCO World Heritage Site

In contrast, Masjid al-Haram in Mecca, Saudi Arabia—Islam’s holiest mosque with origins in the seventh century but continuously expanded—has incorporated sustainability features on a massive scale to accommodate millions of worshippers^23^. While both mosques utilize solar energy and efficient lighting systems, Masjid al-Haram’s sustainability focus emphasizes advanced water recycling systems necessary for its desert climate and enormous visitor numbers^24^. Its approach to sustainability has been more adaptive and retrofitted due to its historical significance and ongoing expansions, while Raja Haji Fisabilillah was designed with sustainability as a founding principle (Table 1).

The Sultan Ahmed Mosque (Blue Mosque) in Istanbul, Turkey (constructed 1609–1617) exemplifies how historical mosques incorporate traditional passive design strategies that preceded modern sustainability concepts. Its classical Ottoman architecture features strategic orientation, natural ventilation, courtyards, and traditional water management systems that have sustained the building for centuries without modern technology^25^. While Raja Haji Fisabilillah employs contemporary technologies, it draws inspiration from these traditional techniques, demonstrating how Islamic architectural heritage can inform modern sustainable design without mere imitation (Table 1).

Perhaps the most striking comparison is with the Great Mosque of Djenné in Mali (current structure from 1907), which represents indigenous sustainability at its most elemental—constructed entirely of adobe bricks with thick walls providing natural insulation in a hot climate^26^. The mosque uses minimal energy, relies on natural cooling, and employs rainwater collection systems. Most notably, the Djenné mosque’s communal maintenance rituals, where the entire community participates in regular replastering, offers a powerful model of social sustainability that complements Raja Haji Fisabilillah’s technological approach. While the Malaysian mosque employs sophisticated green building technologies, the Malian example reminds us that sustainability is also deeply tied to community practices and local material traditions (Table 1).

These comparisons highlight how the Raja Haji Fisabilillah Mosque’s approach represents one path among many within Islamic architectural traditions. What distinguishes this mosque is its explicit certification under contemporary sustainability frameworks (GBI Platinum) while maintaining Islamic design principles—a balance not all mosques have attempted. The mosque’s integration of modern sustainability technologies with Islamic architectural elements demonstrates a distinctive approach to harmonizing faith traditions with contemporary environmental concerns.

The comparative analysis reveals that sustainable mosque design varies significantly based on geographical context, historical period, available resources, and cultural interpretations of Islamic environmental ethics. While some mosques emphasize local materials and passive design (Djenné), others focus on technological solutions (Raja Haji Fisabilillah) or water conservation (Masjid al-Haram). This diversity of approaches underscores that there is no single “Islamic” approach to sustainable design, but rather multiple valid interpretations of how Islamic environmental principles can manifest in architectural form across different contexts.

While the comparative analysis provides a global context for understanding diverse approaches to sustainable mosque design, a closer examination of the specific challenges faced during the Raja Haji Fisabilillah Mosque project offers valuable insights into the practical complexities of implementing these ideals. The process of translating Islamic environmental principles into a functioning, certified green building involved navigating numerous obstacles that required innovative solutions and careful negotiation between competing priorities.

Despite the successful implementation of sustainable features in the Raja Haji Fisabilillah Mosque, the integration process was not without significant challenges. These obstacles provide valuable insights into the complexities of merging contemporary sustainability practices with religious architectural traditions. The project team encountered several interrelated challenges that required innovative solutions and careful negotiation between competing priorities.

Financial considerations presented the first major hurdle. The initial capital investment for sustainable technologies such as solar panels, rainwater harvesting systems, and energy-efficient materials significantly exceeded conventional construction costs. Project stakeholders needed to conduct detailed cost–benefit analyses to demonstrate long-term operational savings that would offset these initial expenses. Additionally, securing funding for innovative green features required extensive education of donors and financial institutions about the alignment between Islamic values of resource stewardship and modern sustainability principles.

Technical challenges emerged as architects and engineers attempted to apply green building standards developed primarily for commercial or residential structures to a religious building with unique functional requirements. The mosque’s need for large, open prayer spaces with specific orientation requirements sometimes conflicted with optimal solar panel placement or natural ventilation configurations. The irregular operational patterns of mosques—with peak usage during prayer times and lower occupancy at other times—required specialized approaches to energy management systems that differed from standard commercial applications.

Cultural and aesthetic considerations presented perhaps the most nuanced challenges. The design team faced the delicate task of balancing innovative sustainable features with traditional mosque aesthetics that would resonate with the local Muslim community. Some stakeholders initially expressed concern that contemporary green design elements might compromise the building’s Islamic character or create a structure that felt unfamiliar to worshippers. Extensive community consultation was necessary to demonstrate how sustainable features could be incorporated while maintaining essential Islamic architectural elements such as minarets, domes, and appropriate ornamentation.

Regulatory hurdles added another layer of complexity. When construction began in 2013, Malaysia’s building codes and Green Building Index (GBI) certification requirements were still evolving for religious structures. The project team often found themselves navigating ambiguous regulatory territory, sometimes pioneering new standards that would later be adopted more broadly. This required close collaboration with regulatory authorities and certification bodies to develop appropriate frameworks for assessing sustainable religious buildings.

Finally, knowledge and skills gaps among local contractors presented practical implementation challenges. Many construction professionals had limited experience with green building technologies, necessitating additional training programs and quality control measures to ensure proper installation and functioning of sustainable systems. This capacity-building effort extended the project timeline but ultimately contributed to raising standards within Malaysia’s construction industry.

Understanding these challenges provides crucial context for appreciating the mosque’s achievements. The obstacles encountered and overcome reveal that integrating sustainability into religious architecture requires not just technical expertise but also cultural sensitivity, theological grounding, community engagement, and institutional commitment. The Raja Haji Fisabilillah Mosque serves as an instructive case study precisely because it navigated these complexities successfully, creating a model that balances innovation with tradition, environmental responsibility with spiritual purpose, and contemporary design with religious identity.

These implementation challenges also highlight important considerations for future sustainable mosque projects globally. They demonstrate that context-specific approaches are essential, as each community must navigate its own unique set of cultural, financial, technical, and regulatory circumstances. The mosque’s experience suggests that extensive stakeholder engagement, careful alignment of sustainability practices with Islamic values, and phased implementation approaches may help overcome similar challenges in other contexts.

A detailed examination of these challenges reveals that sustainable mosque design is not merely a technical endeavor but a complex socio-cultural process requiring negotiation between religious traditions, community expectations, financial constraints, and environmental goals. This fuller understanding of the implementation complexities helps move beyond idealized conceptions of sustainable religious architecture to appreciate the practical pathways through which such visions become reality. The Raja Haji Fisabilillah Mosque’s journey through these challenges offers valuable lessons for religious institutions worldwide seeking to integrate environmental responsibility with spiritual practice in ways that remain authentic to their theological foundations.

## Conclusion

In conclusion, the Raja Haji Fisabilillah Mosque serves as an exemplary paradigm, harmonizing the domains of sustainable development and Islamic architecture. The mosque, through its innovative design, has successfully translated the Islamic principles of stewardship and conservation into tangible, eco-friendly practices. The integration of advanced rainwater harvesting and natural ventilation systems within its structure underpins a commitment to environmental sustainability that extends far beyond the immediate boundaries of the mosque.

The proactive efforts in ecological restoration post-construction, evidenced by the improved Fractional Vegetation Cover (FVC), reflect the mosque’s dedication to not just preserve but enhance the biodiversity of its surroundings. This initiative exemplifies the mosque’s role as a community leader in sustainability, advocating for a balance between development and nature preservation in accordance with Islamic teachings.

Furthermore, the Raja Haji Fisabilillah Mosque has effectively demonstrated how religious structures can act as catalysts for sustainable development within a community. By intertwining faith with environmental stewardship, the mosque exemplifies the Islamic principle of ‘mizan’ (balance) described in Surah Ar-Rahman (55:7–9), where Allah established balance in nature that humans must maintain. The mosque’s harmonization of modern sustainable technologies with traditional Islamic values creates a living model of ‘tawhid’ (unity) applied to environmental ethics, where spiritual practice and ecological responsibility become inseparable aspects of worship (‘ibadah). This approach highlights the potential for religious institutions to foster a culture of ecological consciousness and action, resonating with both Islamic environmental ethics and the global imperative of sustainable development.

This case study underscores the mosque’s dual role as both a place of worship and a beacon of sustainability, setting a benchmark for future religious and secular buildings alike. It serves as a vital demonstration of the role that faith can play in promoting a greener future, positioning the mosque not only as a sanctuary for spiritual reflection but also as a crucible for environmental responsibility.

The Raja Haji Fisabilillah Mosque case study illuminates how religious and cultural motivations can provide powerful foundations for sustainable development initiatives. When environmental practices are understood not as external impositions but as expressions of core religious values, they gain deeper resonance and longevity within communities. The mosque demonstrates that sustainable architecture in religious contexts is most effective when it emerges organically from theological principles and cultural traditions, rather than being merely added as technological solutions.

Looking to the future, this study raises important questions about the evolution of sustainable religious architecture across different faith traditions. How might Christian churches, Jewish synagogues, Hindu temples, or Buddhist monasteries develop their own distinctive approaches to sustainable design that authentically reflect their theological perspectives? What innovations might emerge as various religious traditions confront similar environmental challenges through the lens of their unique spiritual frameworks? Can interreligious dialogue on sustainable architecture foster cross-pollination of ideas and technologies while respecting theological differences?

Additionally, as religious buildings often serve as community anchors with multigenerational influence, they possess unique potential to normalize sustainable practices among their adherents. Future research might explore how sustainable religious buildings can catalyze broader behavioral changes in their surrounding communities, potentially amplifying their environmental impact far beyond their physical footprints.

In this light, the Raja Haji Fisabilillah Mosque encapsulates the essence of sustainable development within the Islamic world, paving the way for a synthesis of spirituality and environmental ethics that is both inspiring and necessary for our times. As climate challenges intensify globally, religious architecture may well become an increasingly important site of environmental innovation, where ancient wisdom and modern technology converge to address the pressing ecological concerns of the twenty-first century.

## Data Availability

All data will be made available on request, please contact the first author Haoxuan Yu (Haoxuan.Yu@monash.edu).
